# Evaluation of the Maximum Velocity of Blood Flow in Descending Aorta in Athletes

**DOI:** 10.3390/jcm15114378

**Published:** 2026-06-05

**Authors:** Georgios A. Christou, Dimitrios N. Kiortsis

**Affiliations:** Department of Radiology, Faculty of Medicine, University of Ioannina, 45332 Ioannina, Greece

**Keywords:** sports cardiology, echocardiography, aorta, coarctation of aorta, athletes

## Abstract

**Background/Objectives:** Athletes are characterized by distinct haemodynamic adaptations of the cardiovascular system, including descending aorta haemodynamics, that could influence the diagnosis of coarctation of the aorta. This study aims to evaluate the normal range for the maximum velocity of blood flow in the descending aorta (Vmax-AoDesc) and the predictors of Vmax-AoDesc in apparently healthy athletes without coarctation of the aorta. **Methods:** We examined 559 asymptomatic healthy athletes with an age of at least 12 years and a tricuspid aortic valve (420 males, age: 29 ± 14 years). We performed evaluations of athletic history, measurements of brachial systolic and diastolic blood pressure, cardiac and aorta ultrasonography and cardiopulmonary exercise testing. Forty athletes were reassessed after a median follow-up of 3.0 (IQR: 2.1) years. **Results:** The median Vmax-AoDesc was 1.29 (IQR: 0.28) m/s, with a maximum of 2.00 m/s. The Vmax-AoDesc could be independently predicted by age (β = −0.392, *p* < 0.001), ratio of systole/diastole (β = 0.095, *p* = 0.023), brachial systolic blood pressure (β = 0.251, *p* < 0.001), left ventricular stroke volume (β = 0.256, *p* < 0.001), ascending aorta diameter (β = −0.230, *p* < 0.001), aortic arch diameter (β = −0.111, *p* = 0.044) and descending aorta diameter (β = −0.103, *p* = 0.017). Age accounted for the greatest variability of Vmax-AoDesc (5.8%). Vmax-AoDesc correlated positively with h/week of endurance exercise training (rho = 0.182, *p* < 0.001) and oxygen uptake at second ventilatory threshold (rho = 0.299, *p* = 0.001). Vmax-AoDesc did not change significantly during follow-up (*p* = 0.438). The median change in Vmax-AoDesc was −0.05 (IQR: 0.18) m/s. However, when Vmax-AoDesc was adjusted for all the above-mentioned independent predictors of Vmax-AoDesc apart from age and systolic blood pressure, there was a reduction in adjusted Vmax-AoDesc during follow-up (*p* = 0.007), indicating a reduction in Vmax-AoDesc with aging. **Conclusions:** The upper limit of the normal range for Vmax-AoDesc was 2.00 m/s in athletes without coarctation of the aorta. Young age was the most important predictor for the measurement of high Vmax-AoDesc. There was an upregulation of Vmax-AoDesc in athletes with a greater volume of endurance exercise training.

## 1. Introduction

Coarctation of the aorta represents a rare congenital narrowing of the thoracic aorta that, if left unrepaired, may lead to serious complications, including severe hypertension, heart failure and aortic dissection [1]. Although coarctation of the aorta is usually diagnosed in infancy or early childhood, there is a possibility of an asymptomatic individual with coarctation of the aorta remaining undiagnosed until adulthood [2,3,4,5,6,7]. Indeed, there are case reports of elderly individuals that were first diagnosed with coarctation of the aorta [2,4,5]. Furthermore, there are case reports of adult athletes with undiagnosed coarctation of the aorta who were previously asymptomatic for many years [5,6,7]. The delayed diagnosis of coarctation of the aorta in these cases may be attributed to a well-developed collateral circulation that possibly allows a preserved distal blood flow and achievement of high levels of functional capacity [5]. Thus, the correct diagnosis of aortic coarctation is of paramount importance in athletes, even in asymptomatic older individuals.

Furthermore, athletes are characterized by distinct haemodynamic adaptations of the cardiovascular system, including downregulation of the heart rate (HR), enhanced stroke volume (SV) and dilatation of cardiac chambers and the aorta [8,9,10,11]. The altered haemodynamics of the cardiovascular system in athletes may affect aorta haemodynamics, with a potential impact on the maximum velocity of blood flow in the descending aorta (Vmax-AoDesc), which is used for the echocardiographic diagnosis of coarctation of the aorta. Thus, the upper limit of normal for Vmax-AoDesc may be substantially different in athletes, thereby influencing the accuracy of diagnosis of coarctation of the aorta. However, Vmax-AoDesc has not been investigated before in athletes.

This study aims to investigate the upper limit of normal for Vmax-AoDesc and the physiological predictors of Vmax-AoDesc in apparently healthy athletes without coarctation of the aorta.

## 2. Materials and Methods

### 2.1. Subjects

In the present cross-sectional study, 559 asymptomatic healthy athletes were consecutively enrolled during pre-participation cardiac screening in order to investigate the upper limit of normal for Vmax-AoDesc and the physiological predictors of Vmax-AoDesc. The inclusion criteria were age of at least 12 years and a tricuspid aortic valve. Exclusion criteria were any cardiac disease, coarctation of the aorta and use of performance enhancing drugs (on the basis of history taking and clinical examination for signs suggestive of concealed abuse of performance enhancing drugs) [12]. In athletes older than 35 years, the diagnosis of coronary artery disease was excluded with the aid of exercise testing or cardiac computed tomography [13].

A medical and athletic history was obtained, including detailed information about the training regimen and training age. All athletes underwent measurement of body weight and height, manual measurement of brachial systolic (SBP) and diastolic (DBP) blood pressure using a sphygmomanometer, a resting electrocardiogram, and transthoracic echocardiography. Moreover, 140 athletes underwent cardiopulmonary exercise testing. Furthermore, 40 athletes were reevaluated prospectively with a second echocardiogram during follow-up.

All participants signed an informed consent document. The current study was carried out according to the Declaration of Helsinki. This study was approved by the Research Ethics Committee of University of Ioannina (13680/2024).

### 2.2. Echocardiography

All echocardiographic images were taken by an experienced cardiologist using a cardiac ultrasound system (Vivid I, GE Medical, GE HealthCare, Chicago, IL, USA) with a 1.5–4.0 MHz phased array transducer. An assessment of the structure and function of the left heart and of the diameters of thoracic aorta was undertaken in agreement with the guidelines of the European Association of Cardiovascular Imaging [14,15]. The left ventricular end-diastolic and end-systolic volumes were measured using the Simpson biplane method. The SV was derived using the equation left ventricular end-diastolic volume − left ventricular end-systolic volume. The diameter of the ascending aorta (AoAsc) was measured in the parasternal long axis view. The diameters of the aortic arch (AoArch) and descending thoracic aorta (AoDesc) were measured in the suprasternal view.

### 2.3. Cardiopulmonary Exercise Testing

Athletes were subjected to cardiopulmonary exercise testing under continuous 12-lead electrocardiographic monitoring until exhaustion on a treadmill using the BRUCE protocol as previously described [16].

The following body weight-indexed spiroergometric parameters were measured: maximum oxygen uptake (VO_2_max), oxygen uptake at first ventilatory threshold (VO_2_-VT1) and oxygen uptake at second ventilatory threshold (VO_2_-VT2).

### 2.4. Statistical Analysis

All statistical analyses were carried out using the software IBM SPSS Statistics 28.0. The missing data were handled by applying Pairwise Deletion. We used the Kolmogorov–Smirnov test to verify the normality of distributions of the studied parameters. Parameters characterized by normal distribution were expressed as mean ± standard deviation, whereas parameters with non-normal distribution were reported as median [interquartile range (IQR)] with the minimum and maximum values. We applied the Mann–Whitney U test to compare the continuous variables between two independent groups. The Kruskal–Wallis H test was used to compare Vmax-AoDesc between the four sport classification categories. Spearman’s correlation analysis, which assessed the univariate associations between the studied parameters, was preceded by analysis for the exclusion of outliers using Cook’s Distance (values characterized by Cook’s Distance > 4/n were regarded as outliers, with n representing the number of observations). We performed prospective validation of post hoc threshold effects when correlation analysis indicated that there were specific cut-offs of the independent variable considerably influencing the correlation coefficient. Specifically, a data-splitting strategy was utilized. Prior to correlation analysis, the total dataset was randomly divided into a 70% training cohort (for threshold discovery) and a 30% independent testing cohort (for validation).

We performed multivariate regression analysis in order to detect the variables with independent associations. The standardized β coefficient was mentioned for each independent variable, as well as the percentage of variance explained by each independent variable, which was derived by squaring the semipartial correlations for each independent variable once the model was finalized. The variance inflation factor (VIF) was used to measure the severity of multicollinearity in multiple regression analysis. A VIF > 10.0 was considered indicative of problematic multicollinearity affecting the regression model. We performed automatic linear modeling as an alternative method of backward stepwise multivariate linear regression analysis, using as a selection method the best subsets entry based on the corrected Akaike information criterion. The LOESS (Locally Estimated Scatterplot Smoothing) Curve was used in scatterplots to reveal and visualize trends. Based on a relevant published study, the inclusion of 36 participants would be expected to result in 80% statistical power to detect a significant association of Vmax-AoDesc with age in a multivariate linear regression model with seven independent variables [17]. However, according to Green’s formula [104 + k (k: number of independent variables)], a minimum sample of 111 participants was needed for multivariate linear regression analysis with seven independent variables. A two-tailed *p* value < 0.05 was regarded as statistically significant. Aiming to adjust for multiple comparisons in the correlation analysis, the original *p* values for correlation analysis were assessed if they were <0.05/18 = 0.003, since there were 18 pairs of variables subjected to correlation analysis.

## 3. Results

### 3.1. Demographic and Athletic Characteristics

The participants included 420 males and 139 females. With regard to race, there were 558 Caucasians and one Black participant. There were 62 elite athletes, 328 amateur competitive athletes and 169 recreational exercisers. Fifteen athletes engaged in skill, 148 in power, 215 in mixed and 181 in endurance sports. The age of the athletes that engaged in endurance sports was greater compared to the athletes of the other three sport classification categories (*p* < 0.001). Thus, we performed adjustment for age in the subgroup analyses between the four sport classification categories to minimize the effect of any potential selection bias. The mean age was 29 ± 14 years old. The mean training age was 12 ± 9 years. The current training regimen included 6.4 ± 3.8 h of exercise training per week, among which 4.5 ± 4.3 h was endurance exercise training per week.

### 3.2. Normal Range of Vmax-AoDesc

Table 1 shows the Vmax-AoDesc for the whole study population and for the subgroups. The median Vmax-AoDesc was 1.29 (IQR: 0.28) m/s, with a maximum of 2.00 m/s.

### 3.3. Demographic Predictors of Vmax-AoDesc

Vmax-AoDesc was slightly higher in males compared to females (*p* = 0.042) (Table 1).

**Figure 1:**
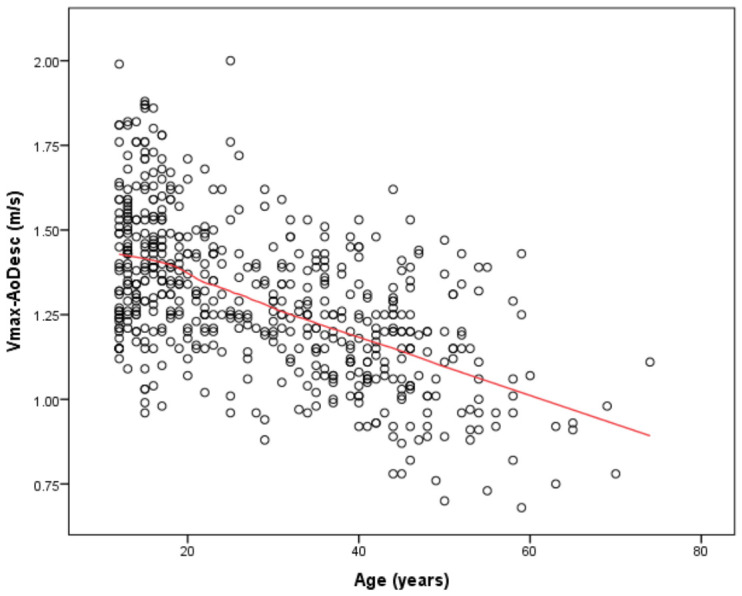
LOESS Curve showing the association between the maximum velocity of blood flow in the descending aorta (Vmax-AoDesc) and age.

Vmax-AoDesc correlated negatively with age (rho = −0.573, *p* < 0.001). Specifically, this association was evident only in athletes with an age > 20 years (rho = −0.487, *p* < 0.001), since this association was nonsignificant for athletes with an age ≤ 20 years. These results are demonstrated visually in Figure 1, which shows the LOESS Curve of the association between Vmax-AoDesc and age. This cut-off of age was confirmed through the prospective validation of post hoc threshold effects in both cohorts, derived after a random data-splitting strategy.

Table 2 shows the age range of the athletes with Vmax-AoDesc greater than or equal to a specific cut-off value. The maximum age of the athletes increased as the cut-off value of Vmax-AoDesc decreased. Notably, the older athlete with Vmax-AoDesc ≥ 1.7 m/s was 26 years old, the older athlete with Vmax-AoDesc ≥ 1.5 m/s was 46 years old, and the oder athlete with Vmax-AoDesc ≥ 1.2 m/s was 59 years old.

### 3.4. Haemodynamic Predictors of Vmax-AoDesc

**Figure 2:**
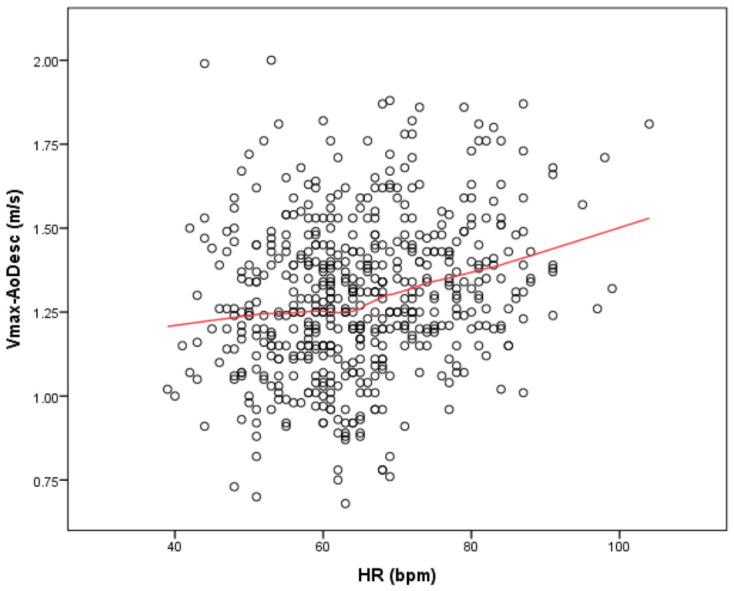
LOESS Curve showing the association between the maximum velocity of blood flow in the descending aorta (Vmax-AoDesc) and heart rate (HR).

Vmax-AoDesc was associated positively with HR (rho = 0.241, *p* < 0.001), the duration of systole (rho = 0.104, *p* = 0.028) and the ratio of systole/diastole (rho = 0.287, *p* < 0.001) and correlated negatively with the duration of diastole (rho = −0.265, *p* < 0.001). We performed the enter method of multivariate linear regression analysis to predict Vmax-AoDesc using the duration of systole (β = 0.107, *p* = 0.017) and duration of diastole (β = −0.245, *p* < 0.001) as independent variables. In this analysis, the duration of diastole (6.0%) accounted for greater variability of Vmax-AoDesc compared to the duration of systole (1.2%). Moreover, we used the enter method of multivariate linear regression analysis to predict Vmax-AoDesc using HR (β < 0.001, *p* = 0.998) and the ratio of systole/diastole (β = 0.285, *p* < 0.001) as independent variables. In this analysis, the ratio of systole/diastole (2.7%) accounted for greater variability of Vmax-AoDesc compared to the HR (<0.01%). The VIF was 3.0 for both independent variables, indicating no problematic multicollinearity affecting the regression model. With regard to the relationship between Vmax-AoDesc and HR, this association was evident only for HR ≥ 65 bpm (rho = 0.247, *p* < 0.001), since this association was nonsignificant for HR < 65 bpm. These results are demonstrated visually in Figure 2, which shows the LOESS Curve of the association between Vmax-AoDesc and HR. This cut-off of HR was confirmed through the prospective validation of post hoc threshold effects in both cohorts, derived after a random data-splitting strategy.

Vmax-AoDesc correlated positively with SBP (rho = 0.120, *p* = 0.011), and this association persisted after adjustment for age. However, there was not any significant association between Vmax-AoDesc and DBP after adjustment for age.

Although Vmax-AoDesc was not significantly associated with SV in univariate analysis, a positive association (β = 0.196, *p* < 0.001) emerged after adjustment for age.

### 3.5. Anatomic Predictors of Vmax-AoDesc

Vmax-AoDesc correlated negatively with AoAsc (rho = −0.486, *p* < 0.001), AoArch (rho = −0.417, *p* < 0.001) and AoDesc (rho = −0.197, *p* < 0.001).

### 3.6. Athletic Characteristics Predicting Vmax-AoDesc

There was no difference in the Vmax-AoDesc between the four sport classification categories (*p* = 0.548) after adjustment for age (Table 1). Vmax-AoDesc was not significantly associated with training age (*p* = 0.973) after adjustment for age.

**Figure 3:**
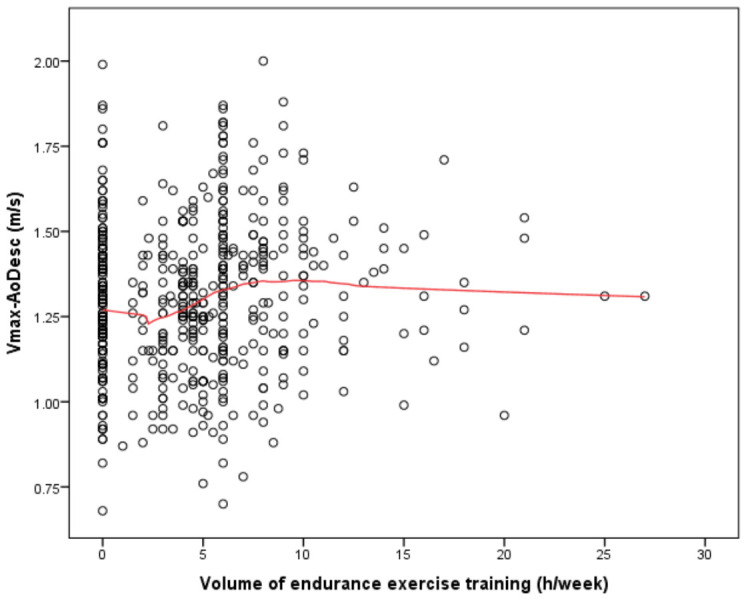
LOESS Curve showing the association between the maximum velocity of blood flow in the descending aorta (Vmax-AoDesc) and volume of endurance exercise training.

The Vmax-AoDesc correlated positively with h/week of exercise training (rho = 0.158, *p* < 0.001). When we performed the enter method of multivariate linear regression analysis to predict Vmax-AoDesc using the following two independent variables, h/week of endurance exercise training and h/week of nonendurance exercise training (i.e., the two components of exercise training), the only independent predictor of Vmax-AoDesc was h/week of endurance exercise training (β = 0.105, *p* = 0.042). Therefore, the association between Vmax-AoDesc and h/week of exercise training was attributed to the association between Vmax-AoDesc and h/week of endurance exercise training (rho = 0.182, *p* < 0.001). Specifically, the association between Vmax-AoDesc and h/week of endurance exercise training was evident only in the range of 2–8 h/week of endurance exercise training (rho = 0.266, *p* < 0.001), since this association was nonsignificant for ≤2 or >8 h/week. These results are demonstrated visually in Figure 3, which shows the LOESS Curve of the association between Vmax-AoDesc and h/week of endurance exercise training. These cut-offs of the volume of endurance exercise training were confirmed through prospective validation of post hoc threshold effects in both cohorts, derived after a random data-splitting strategy. After using the enter method of multivariate linear regression analysis in the athletes with 2–8 h/week of endurance exercise training, the association between Vmax-AoDesc and h/week of endurance exercise training (β = 0.130, *p* = 0.011) persisted even after adjustment for age, whereas this association disappeared after further adjustment for SV.

Vmax-AoDesc correlated positively with VO_2_max (rho = 0.184, *p* = 0.035) and VO_2_-VT2 (rho = 0.299, *p* = 0.001), but not with VO_2_-VT1. Therefore, among these spiroergometric variables, Vmax-AoDesc correlated more strongly with VO_2_-VT2. After performing the enter method of multivariate linear regression analysis, the association between Vmax-AoDesc and VO_2_-VT2 (β = 0.156, *p* = 0.035) persisted even after adjustment for age.

### 3.7. Integrative Analysis of the Predictors of Vmax-AoDesc

Taking into account that SV was greater in males compared to females (*p* < 0.001), in order to investigate the influence of sex on Vmax-AoDesc, we compared Vmax-AoDesc between males and females after adjustment for age and SV using the enter method of multivariate linear regression analysis. Vmax-AoDesc did not differ between males and females after adjustment for age and SV either in the subgroup of athletes aged ≥16 years or in the subgroup of athletes aged ≤14 years. Therefore, sex was not considered an important independent predictor of Vmax-AoDesc.

We used backward stepwise multivariate linear regression analysis to predict Vmax-AoDesc using age, the ratio of systole/diastole, SBP, SV, AoAsc, AoArch and AoDesc as independent variables. Therefore, in the final model, the Vmax-AoDesc could be independently predicted by age (β = −0.392, *p* < 0.001, 5.8% of variability), the ratio of systole/diastole (β = 0.095, *p* = 0.023, 0.7% of variability), SBP (β = 0.251, *p* < 0.001, 4.8% of variability), SV (β = 0.256, *p* < 0.001, 3.8% of variability), AoAsc (β = −0.230, *p* < 0.001, 1.8% of variability), AoArch (β = −0.111, *p* = 0.044, 0.6% of variability) and AoDesc (β = −0.103, *p* = 0.017, 0.8% of variability). The final model was characterized by an adjusted R^2^ = 0.454; *p* < 0.001. The VIF was <3.0 for all independent variables, indicating no problematic multicollinearity affecting the regression model. Moreover, we used automatic linear modeling as an alternative method of backward stepwise multivariate linear regression analysis, which showed that the Vmax-AoDesc could be predicted by the following independent variables in decreasing order of importance: age (44% importance), SV (23% importance), SBP (15% importance), ratio of systole/diastole (8% importance), AoAsc (6% importance), AoDesc (2% importance) and AoArch (2% importance).

### 3.8. Changes in Vmax-AoDesc During Follow-Up

Vmax-AoDesc did not change significantly (*p* = 0.438) after a median follow-up of 3.0 (IQR: 2.1) years. The median change in Vmax-AoDesc was −0.05 (IQR: 0.18) m/s. We created the composite variable of Vmax-AoDesc adjusted for all the above-established independent predictors of Vmax-AoDesc in multivariate analysis apart from age and SBP. The formula for this calculation was as follows: adjusted Vmax-AoDesc = Vmax-AoDesc × AoAsc × AoArch × AoDesc/(HR × SV). Thus, the adjusted Vmax-AoDesc decreased during follow-up (*p* = 0.007), indicating a reduction in Vmax-AoDesc with aging.

## 4. Discussion

The key findings of the present study include the following: (1) the upper limit of normal for Vmax-AoDesc was 2.00 m/s in athletes without aortic coarctation; (2) Vmax-AoDesc could be independently predicted by age, ratio of systole/diastole, SBP, SV and thoracic aortic diameters, with age accounting for the greatest variability of Vmax-AoDesc; (3) Vmax-AoDesc was upregulated in athletes with a greater volume of endurance exercise training.

### 4.1. The Upper Limit of Normal for Vmax-AoDesc

The current study found that the upper limit of normal for Vmax-AoDesc was 2.00 m/s for athletes with an age of at least 12 years old. Therefore, an echocardiographic measurement of Vmax-AoDesc > 2.00 m/s in an athlete could raise suspicion for the presence of coarctation of the aorta, particularly when there is coexistent hypertension. The enhancement of Vmax-AoDesc in athletes with an increased volume of endurance exercise training that was demonstrated in the present study indicates that nonathletes are possibly characterized by lower Vmax-AoDesc compared to athletes, and thus the criterion of “Vmax-AoDesc > 2.00 m/s” as a potential sign of cardiovascular abnormality may be even more valid for nonathletes [18]. In this respect, a cut-off of 2.00 m/s for Vmax-AoDesc could be used for nonathletes as a diagnostic criterion with higher specificity compared to athletes. To the best of our knowledge, no previous study has investigated the upper limit of normal for Vmax-AoDesc in athletes. Moreover, the upper limit of normal for Vmax-AoDesc has not been reported in adult nonathletes so far, since the few relevant studies that included Vmax-AoDesc in their results did not mention the upper bound of the observed range of Vmax-AoDesc [18,19].

### 4.2. The Age as a Predictor of Vmax-AoDesc

The most important independent predictor of Vmax-AoDesc in athletes that was shown in our study was chronological age, implying that the detection of high Vmax-AoDesc in elderly individuals renders the diagnosis of coarctation of the aorta highly likely. Specifically, the measurement of Vmax-AoDesc ≥ 1.2 m/s in athletes with an age of at least 60 years should be regarded as an abnormal finding needing further investigation. Furthermore, the detection of Vmax-AoDesc ≥ 1.5 m/s in athletes with an age of at least 50 years could represent an abnormal finding as well. All these considerations are particularly true when there is coexistent abnormally high blood pressure. The negative association of Vmax-AoDesc with age could be plausibly explained by the reduction in distensibility of the aorta with aging, which in turn may increase the aortic impedance and thus could induce a reduction in forward blood flow through the aorta compared to the blood flow that is directed from the aorta to its branches [20,21,22]. These considerations are particularly relevant for the aortic arch and its branches. The histological process underlying the reduction in distensibility of the aorta with aging possibly includes the degradation of elastin fibers as a result of the pulsatile stress-induced fraction of elastin fibers with subsequent partial replacement of them by fibrotic tissue [21].

### 4.3. The Ratio of Systole/Diastole as a Predictor of Vmax-AoDesc

We demonstrated that Vmax-AoDesc was upregulated in situations with high HR or a high ratio of systole/diastole, with the latter being the most important independent predictor among them. Notably, the duration of diastole was shown to be more important for the determination of Vmax-AoDesc than the duration of systole. The underlying mechanism for these findings may be the fact that the relative increase in the duration of diastole is expected to enhance the redistribution of the blood volume from the proximal aorta to the descending aorta during elastic recoil of the proximal aorta in diastole, resulting in greater blood volume needed to expand the proximal aorta during systole, and by this means leading to a smaller part of SV being available for forward blood flow through the descending aorta [20]. Taking into account that the positive association between Vmax-AoDesc and HR was particularly evident in athletes with HR > 65 bpm, the measurement of Vmax-AoDesc could be reasonably suggested to be performed in situations with HR up to 65 bpm, allowing a minimization of HR-induced regulation of Vmax-AoDesc and thus higher levels of standardization in Vmax-AoDesc measurements.

### 4.4. The SV as a Predictor of Vmax-AoDesc

The positive association of Vmax-AoDesc with SV is possibly interpreted on the basis of the fact that Vmax-AoDesc is proportional to the blood flow through the descending aorta, since the latter represents a considerable part of the blood flow that is ejected from the heart to the thoracic aorta. The higher the elasticity of the thoracic aorta, the higher the part of SV directed to the aorta rather than to the aortic branches, permitting an increased part of SV available for forward blood flow through the descending aorta [20]. Consistently, the association between Vmax-AoDesc and SV was considerably strengthened after adjustment for age as a proxy measure of elasticity of the aorta.

### 4.5. The Brachial SBP as a Predictor of Vmax-AoDesc

The positive association of Vmax-AoDesc with brachial SBP in the present study may be accounted for by the decrease in pressure gradient between the aortic arch and the arteries of the upper arms in individuals with higher SBP. This downregulation of the pressure gradient could lead to a relative reduction in blood flow directed to the arteries of the upper arms in individuals with higher SBP, resulting in enhanced forward blood flow through the aortic arch and downstream through the descending aorta. The possible augmentation of Vmax-AoDesc when the brachial SBP is increased implies that the correct assessment of Vmax-AoDesc can reasonably be preceded by the control of SBP.

### 4.6. The Aortic Diameters as Predictors of Vmax-AoDesc

The current study found for the first time a negative association of Vmax-AoDesc with AoAsc, AoArch and AoDesc that could be explained, first, on the basis of the increase in turbulent blood flow as thoracic aortic diameters increase, with a resulting loss of kinetic energy, which dissipates into heat, and, secondly, due to the fact that the mean forward blood velocity (i.e., average spatial velocity across a cross-section) through the aorta equates to the blood flow through the aorta/cross-sectional area of the aorta [23,24]. The progressive dilatation of the thoracic aorta with aging is expected to contribute to the accompanied decline in Vmax-AoDesc as an individual becomes older [25]. Based on the aforementioned considerations, in athletes with dilatation of the thoracic aorta, there may be a higher possibility of false negative echocardiographic diagnosis of coarctation of the aorta.

### 4.7. The Volume of Endurance Exercise Training as a Predictor of Vmax-AoDesc

Importantly, the present study demonstrated an enhancement of Vmax-AoDesc in athletes with a greater volume of endurance exercise training. To the best of our knowledge, no previous study has investigated Vmax-AoDesc in athletes. The leading cause of the positive association between Vmax-AoDesc and the volume of endurance exercise training is possibly the augmentation of SV in endurance-trained athletes, as implied by the results of adjusted analysis, while the commonly encountered low HR and relatively increased thoracic aortic diameters in endurance-trained athletes tends to weaken this association [9,26,27]. These competing mechanisms of increased SV from one side tending to increase Vmax-AoDesc and both low HR and increased thoracic aortic diameters from the other side tending to reduce Vmax-AoDesc may explain the fact that the positive association between Vmax-AoDesc and the volume of endurance exercise training was particularly evident in the intermediate range of 2–8 h/week of endurance exercise training. The fact that Vmax-AoDesc correlated more strongly with VO_2_-VT2 than VO_2_max further reinforces the concept of the association between Vmax-AoDesc and volume of endurance exercise training, since the increase in the volume of endurance exercise training is known to enhance more performance at VT2 than VO_2_max [9].

### 4.8. Study Strengths and Limitations

The strengths of the current study include, firstly, the fact that we investigated for the first time Vmax-AoDesc in apparently healthy athletes without coarctation of the aorta. Secondly, we assessed Vmax-AoDesc in a large number of athletes practicing various sports belonging to the four sport classification categories Moreover, we explored in an integrative manner all the potential determinants of Vmax-AoDesc, evaluating the relative importance of each predictor, and we investigated the independent contribution of each predictor, attempting to shed light on the underlying mechanisms of the associations.

The results of the present study should be interpreted in light of the following limitations. Firstly, the total number of skill and power athletes was smaller than the total number of mixed and endurance athletes, resulting in decreased power of this study to detect significant differences between these groups. Secondly, the age of the athletes that engaged in endurance sports was greater compared to the athletes of the other three sport classification categories. Nevertheless, we performed adjustment for age in the subgroup analyses between the four sport classification categories to minimize the effect of any potential selection bias. Although the design of the study for the whole study population was cross-sectional, leading to a decreased ability of firm conclusions to be drawn about causality, a subgroup of athletes was examined prospectively. Fourthly, even though the missing data were handled by applying Pairwise Deletion, in which the sample size can vary across different variable pairs of correlation analysis, this methodology is associated with a maximization of sample size for each unique parameter calculation along with an enhancement of statistical power. An additional limitation of the current study represents the lack of external validation. Thus, further studies are needed to confirm our results. Moreover, among the athletes that were examined prospectively, there were no follow-up measurements of SBP available for analysis. Furthermore, the suprasternal echocardiographic measurement of AoDesc classically allows only a partial visualization of the descending aorta and may not estimate the true lumen diameter accurately enough [28].

## 5. Conclusions

In conclusion, we found that the upper limit of normal for Vmax-AoDesc was 2.00 m/s in our cohort of athletes without coarctation of the aorta. The most important independent predictor of Vmax-AoDesc in athletes may be chronological age, while an upregulation of Vmax-AoDesc could be normally expected in athletes with a greater volume of endurance exercise training, though there were some inequalities in the sample size and age between the athletes of the four sport classification categories. The inclusion of these considerations in the diagnostic work-up of athletes subjected to preparticipation screening, particularly those with high blood pressure, could enable a more precise and standardized assessment of Vmax-AoDesc and thus enhance the accuracy of the diagnostic work-up for coarctation of the aorta.

## Figures and Tables

**Table 1:** The maximum velocity of blood flow in descending aorta (Vmax-AoDesc) in the whole study population and in the subgroups.

	Vmax-AoDesc (m/s)	IQR (m/s)	Range (m/s)
**All**	1.29	0.28	0.68–2.00
**Males**	1.29	0.30	0.70–2.00
**Females**	1.25	0.28	0.68–1.81
**Skill sports**	1.25	0.12	0.93–1.43
**Power sports**	1.25	0.28	0.68–1.99
**Mixed sports**	1.38	0.29	0.82–2.00
**Endurance sports**	1.21	0.29	0.70–1.88

Range is expressed as minimum–maximum. **Abbreviations:** IQR: interquartile range, Vmax-AoDesc: maximum velocity of blood flow in descending aorta.

**Table 2:** The age range of the athletes with maximum velocity of blood flow in descending aorta (Vmax-AoDesc) greater than or equal to a specific cut-off value.

Vmax-AoDesc (m/s)	Age Range (Years)
≥1.9	12–25
≥1.8	12–25
≥1.7	12–26
≥1.6	12–44
≥1.5	12–46
≥1.4	12–59
≥1.3	12–59
≥1.2	12–59
≥1.1	12–74
≥1.0	12–74
≥0.9	12–74
≥0.8	12–74
≥0.7	12–74

Age range is expressed as minimum–maximum. **Abbreviations.** Vmax-AoDesc: maximum velocity of blood flow in descending aorta.

## Data Availability

Dataset available on reasonable request from the authors.
